# Happiness, Life Satisfaction and Emotional Exhaustion Among University Students in Spain and Germany: Exploratory Between-Sample Comparisons

**DOI:** 10.3390/ejihpe16090135

**Published:** 2026-09-08

**Authors:** Ana María Morales-Rodríguez, Francisco Manuel Morales-Rodríguez

**Affiliations:** 1 Department of Journalism, Faculty of Communication Sciences, University of Málaga, 29010 Malaga, Spain; 2 Department of Educational and Developmental Psychology, Faculty of Psychology, University of Granada, 18011 Granada, Spain

**Keywords:** well-being, happiness, life satisfaction, burnout, university students

## Abstract

University students are increasingly exposed to academic and emotional demands associated with burnout and psychological distress in contemporary higher education contexts. Objective: This study examined academic-context well-being indicators, multidimensional happiness and global life satisfaction among university students in Spain and Germany, as well as their associations with burnout and compassion-related variables. Methods: A quantitative, cross-sectional study with exploratory between-sample comparisons was conducted using a convenience sample of university students from both countries. Measures assessing academic-context well-being indicators, global life satisfaction, multidimensional happiness, burnout-related dimensions and compassion-related variables were administered. Analyses included descriptive statistics, Pearson correlations, gender comparisons and exploratory between-sample observed-score comparisons using Welch’s independent-samples *t*-tests with Holm adjustment for multiple comparisons. Results: Descriptive findings indicated positive scores on several well-being indicators alongside burnout-related strain. Global life satisfaction was positively associated with dimensions of happiness and inversely associated with burnout-related indicators. No significant gender differences were observed. After Holm adjustment, statistically significant observed-score differences between the two samples remained for 10 of the 17 indicators examined. The largest differences were observed for compassion satisfaction, ProQOL burnout and expected job satisfaction. Conclusions: The exploratory comparisons indicated a domain-specific pattern of observed-score differences between the Spanish and German samples rather than a general well-being advantage for either group. The results highlight the relevance of promoting student well-being and addressing burnout-related strain while considering the contextual characteristics of different university settings.

## 1. Introduction

The study of well-being, happiness and mental health among university students has become increasingly important in educational psychology and the social sciences. Theoretical approaches to well-being encompass both hedonic and eudaimonic perspectives (Ryan & Deci, 2001), while demand–resource models emphasise the role of contextual conditions in shaping well-being and psychological strain (Bakker & Demerouti, 2017). In Europe, recent reports on living conditions, mental health and the effects of the pandemic on young people underscore the importance of studying emerging psychosocial risks and the accelerated transformations of the educational environment (Eurofound, 2021).

Subjective well-being is commonly conceptualised as involving cognitive and affective evaluations of one’s life (Diener et al., 1999). Within this framework, global life satisfaction refers to a cognitive appraisal of life as a whole and is assessed in the present study using the Satisfaction With Life Scale (SWLS) (Diener et al., 1985). Happiness is examined using the Lima Happiness Scale (LHS), a multidimensional measure originally structured into four dimensions: positive sense of life, life satisfaction, personal fulfilment and joie de vivre (Alarcón, 2006). The SWLS and LHS are conceptually related and partially overlapping rather than fully independent measures. The SWLS provides a focused global evaluation of life satisfaction, whereas the LHS assesses a multidimensional profile of happiness in which life satisfaction constitutes one of four dimensions. Accordingly, the two measures are treated in the present study as operationally distinct, while acknowledging their conceptual overlap.

In the context of higher education, students face a particular set of academic and emotional demands: excessive workload, pressure from assessments, uncertainty about future employment, and increasing demands for self-regulation. These academic pressures have been associated with student stress and burnout (Lesener et al., 2020; Pascoe et al., 2020).

Well-being in higher education is shaped not only by positive evaluations of life but also by the demands and resources present in the academic environment. The Study Demands–Resources framework conceptualises academic demands and resources as relevant to student burnout and engagement (Lesener et al., 2020), providing a rationale for examining burnout alongside well-being. Evidence from higher-education samples further supports associations between burnout, academic demands, resources and engagement (Morales-Rodríguez et al., 2019; Salmela-Aro & Read, 2017; Zeijen et al., 2024).

Recent developments in Study Demands–Resources theory further conceptualise student well-being through partly distinct but interacting processes: sustained study demands may contribute to strain and burnout, whereas study resources may support motivation, engagement and adaptive functioning (Bakker & Mostert, 2024). This framework therefore provides a theoretical basis for examining burnout-related strain alongside positive indicators of student well-being, while recognising that these dimensions are related but not interchangeable.

These academic conditions are also being reshaped by rapid digitalisation and the increasing integration of artificial intelligence into higher education, creating additional contextual challenges for students’ learning experiences (Bond et al., 2024; European Commission, 2022).

Compassion-related variables were included as complementary indicators of the emotional costs and rewards that may arise in educational and pre-professional activities involving interpersonal or helping demands. Previous research has examined ProQOL-related dimensions in students undertaking helping-oriented professional training, particularly in social work and nursing contexts (Harr et al., 2014; Chachula, 2021; Hamaideh et al., 2023). In the present study, these variables are therefore treated as psychosocial correlates of well-being rather than as interchangeable components of the same construct. Given the heterogeneity of the present sample, their interpretation is exploratory and does not assume equivalence with professional helping contexts; in the present study, item wording was adapted where necessary to refer to the academic context.

Against this background, jointly examining positive well-being indicators, burnout-related dimensions and compassion-related variables may provide a more integrated account of psychosocial experiences among university students across different higher-education contexts.

Although life satisfaction, multidimensional happiness, burnout and compassion-related experiences have been examined in university populations, these dimensions are frequently considered separately or within specific training contexts. Examining them jointly may provide a more differentiated picture of student well-being by showing whether positive evaluations of life can coexist with burnout-related strain rather than assuming that well-being and strain represent opposite ends of a single continuum. Recent empirical evidence supports this multidimensional perspective. Academic burnout has been negatively associated with life satisfaction among university students (Gutierrez Ticona et al., 2024), while person-oriented research has identified distinct study well-being profiles characterised by different combinations of engagement, burnout and study satisfaction (Vilhunen et al., 2026). Together, these findings suggest that student well-being is better understood by considering the configuration of positive and strain-related indicators rather than relying on a single global dimension.

Based on these considerations, the aim of this study was to describe academic-context well-being indicators, multidimensional happiness and global life satisfaction among university students in Spain and Germany, as well as to explore observed-score differences according to gender and country of study. The study also aimed to examine associations among well-being indicators and psychosocial variables linked to academic strain, including burnout and compassion-related variables. By examining students recruited in two European higher-education contexts, this study seeks to contribute to current discussions on student mental health, academic well-being and the psychosocial challenges associated with contemporary higher education systems.

## 2. Materials and Methods

### 2.1. Study Design

A quantitative, cross-sectional, ex post facto correlational design with exploratory between-sample comparisons was employed. This design enabled the description of academic-context well-being indicators, happiness and life satisfaction among university students in Spain and Germany, as well as the examination of associations among psychosocial variables and observed-score differences according to gender and country of study. Because the design was cross-sectional, all associations are interpreted as non-causal.

### 2.2. Participants

The convenience sample comprised 628 university students enrolled in various degree programmes in Spain and Germany. Specifically, the sample included 408 Spanish students and 220 German students. Most participants were enrolled in the first years of their university studies, corresponding to a young university student profile characteristic of late adolescence and emerging adulthood. The gender distribution was balanced, and no substantial imbalances were observed by age. This sample composition permitted exploratory comparisons of well-being, happiness and emotional exhaustion according to gender and country of study. Because convenience sampling was used, the sample size was not determined through an a priori power calculation.

### 2.3. Instruments

The following instruments and adapted indicators were used to assess the study variables. Academic-context well-being indicators were adapted from Hassani (2017). Global life satisfaction was assessed using the Satisfaction With Life Scale (SWLS) (Diener et al., 1985), and happiness using the Lima Happiness Scale (LHS) (Alarcón, 2006). Burnout-related dimensions were assessed using the Maslach Burnout Inventory (MBI) (Maslach & Jackson, 1981; Maslach et al., 1996), whereas compassion satisfaction, burnout and secondary traumatic stress were assessed using the Professional Quality of Life Scale (ProQOL) (Stamm, 2010). All instruments were administered in the language of the respective study sample, with Spanish-language questionnaires used in Spain and German-language questionnaires used in Germany.

The instruments administered are described below:

Academic-context well-being indicators were used; these included six single-item indicators adapted from the questionnaire developed by Hassani (2017) and reformulated for the academic context, which were used to assess students’ subjective perceptions of their academic context: specified activities, autonomy, views on teaching, conditions of the educational institution, passion for the future profession and expected job satisfaction. Each indicator was rated on a 10-point scale, with higher scores representing a more favourable perception of the corresponding academic characteristic. The indicators were analysed separately rather than combined into a total score. The set of indicators showed an internal consistency coefficient of α = 0.91 in the present sample; however, this coefficient is reported descriptively because the indicators were treated as distinct measures rather than as a single unidimensional scale. These indicators are interpreted as subjective perceptions and not as objective evaluations of teaching quality or institutional performance. An example item is as follows: “I have adequate conditions to carry out my academic activities”. Previous studies have reported adequate internal consistency, with Cronbach’s alpha coefficients above 0.80 in European organisational contexts, supporting its use for assessing workplace well-being in professional settings.

We also used the Satisfaction With Life Scale (SWLS). Global life satisfaction was assessed using the Satisfaction With Life Scale (SWLS) (Diener et al., 1985). The scale comprises five items rated from 1 (strongly disagree) to 7 (strongly agree). The five item scores are summed to obtain a total score with a theoretical range of 5–35; higher scores indicate greater global life satisfaction. To distinguish this measure from the life-satisfaction dimension of the Lima Happiness Scale, this variable is consistently labelled “SWLS total score” throughout the manuscript. An example item is as follows: “In most respects, my life is just as I would like it to be”. The original validation study reported high internal consistency (α = 0.87) and a two-month test–retest reliability coefficient of 0.82 (Diener et al., 1985). Subsequent studies have also supported the psychometric adequacy of the Spanish and German versions of the SWLS (Vázquez et al., 2013; Glaesmer et al., 2011). Internal consistency in the present sample was α = 0.86.

Furthermore, we used the Lima Happiness Scale (LHS). Happiness was assessed using the 27-item Lima Happiness Scale (LHS) (Alarcón, 2006), with items rated from 1 (strongly disagree) to 5 (strongly agree). The instrument comprises four dimensions: LHS positive sense of life (11 items; theoretical range 11–55); LHS life satisfaction (6 items; 6–30); LHS personal fulfilment (6 items; 6–30); and LHS joie de vivre (4 items; 4–20). Subscale scores were obtained by summing the corresponding items after the required reverse scoring, with higher scores indicating higher levels of the dimension assessed. An example item is as follows: “I feel satisfied with what I have achieved in my life”. The original validation study, conducted with university students, showed high internal consistency for the total scale (α = 0.916) and adequate reliability for its four dimensions (α = 0.88, 0.79, 0.76 and 0.72, respectively), as well as evidence of convergent and factorial validity (Alarcón, 2006). A subsequent psychometric study in university students also supported the four-factor structure and the adequacy of the scale for this population (Arias Gallegos et al., 2016).

The Maslach Burnout Inventory (MBI) was also used. Burnout-related dimensions were assessed using the 22-item MBI (Maslach & Jackson, 1981; Maslach et al., 1996). Items are rated on a 7-point frequency scale from 0 (never) to 6 (every day). The instrument comprises emotional exhaustion (9 items; theoretical range 0–54), depersonalisation (5 items; 0–30) and personal accomplishment (8 items; 0–48). Higher emotional exhaustion and depersonalisation scores indicate greater strain, whereas higher personal accomplishment indicates greater perceived accomplishment and therefore lower burnout. In the present study, item wording was adapted to the academic context where necessary, replacing references to professional activities with references to students’ studies and academic activities. Internal consistency coefficients for the MBI dimensions in the present sample ranged from α = 0.80 to 0.94. The three dimensions were analysed as continuous variables. An example item is as follows: “I feel emotionally drained by my studies”. The original MBI showed a three-dimensional structure comprising emotional exhaustion, depersonalisation and personal accomplishment, with evidence supporting its reliability and validity (Maslach & Jackson, 1981). The instrument exhibits high internal consistency, with Cronbach’s alpha values of approximately 0.90 for emotional exhaustion, 0.79 for depersonalisation and 0.71 for personal accomplishment (Maslach et al., 1996). Versions of the 22-item MBI retaining these three dimensions have previously been used with university student populations, providing a precedent for its application in academic contexts (Chigerwe et al., 2014).

Finally, we used the Professional Quality of Life Scale (ProQOL). Compassion satisfaction, burnout and secondary traumatic stress were assessed using the 30-item ProQOL (Stamm, 2010). The instrument comprises three 10-item subscales: compassion satisfaction, burnout and secondary traumatic stress. Items are rated on a 5-point scale from 1 to 5. After reverse-scoring the corresponding items in accordance with the scoring manual, the items within each subscale are summed, yielding theoretical raw-score ranges of 10–50. Higher values indicate higher compassion satisfaction, burnout or secondary traumatic stress, respectively. Internal consistency coefficients for the ProQOL dimensions in the present sample ranged from α = 0.80 to 0.92. The three subscales were analysed as continuous variables. An example item is as follows: “I enjoy being able to help other people”. In the present study, item wording was adapted where necessary to refer to the academic context. The instrument demonstrates adequate levels of reliability, with Cronbach’s alpha coefficients of approximately 0.88 for compassion satisfaction, 0.75 for burnout and 0.81 for secondary traumatic stress. The ProQOL manual also reports evidence supporting the construct validity of the instrument and the distinction among its three subscales (Stamm, 2010). Previous studies have applied the ProQOL to university students, including students in helping-oriented and pre-professional training contexts (Chachula, 2021; Hamaideh et al., 2023; Morales-Rodríguez, 2026), providing a precedent for examining these dimensions in student populations. The ProQOL dimensions were treated as continuous psychosocial indicators rather than as diagnostic measures (Stamm, 2010).

### 2.4. Procedure

Data were collected primarily using paper-and-pencil questionnaires administered in person and, where applicable, through a secure online platform. Invitations were circulated through university channels and academic networks in Spain and Germany. Participation was voluntary and anonymous. Before completing the questionnaires, participants received information about the aims of the study, confidentiality and data protection, and their right to withdraw at any time. Written informed consent was obtained from all participants prior to participation. The ethical principles set out in the Declaration of Helsinki and local data protection regulations were adhered to.

### 2.5. Statistical Analysis

Data analyses were conducted using IBM SPSS Statistics for Windows, version 32.0 (IBM Corp., Armonk, NY, USA). Descriptive statistics were calculated for the study variables. Distributional assumptions were examined using skewness and kurtosis indicators. Pearson correlations were used to examine bivariate associations among the principal study variables. Gender differences were examined using independent-samples *t*-tests. Between-country differences were examined using two-sided Welch’s independent-samples *t*-tests because the country groups were unequal in size and equality of variances was not assumed. To control the family-wise error rate across the 17 final between-country comparisons, raw *p* values were adjusted using Holm’s sequential procedure. Cohen’s d and Hedges’ g were reported as standardised effect-size estimates. Statistical significance for the between-country comparisons was evaluated using Holm-adjusted two-sided *p* values < 0.05. These between-sample analyses were exploratory and refer only to observed composite-score differences. In the absence of measurement-invariance testing, they were not interpreted as evidence of latent or invariant cross-cultural differences. The analytical strategy was descriptive and associational; no multivariable predictive models were included, and the reported associations should not be interpreted as independent predictive effects.

## 3. Results

### 3.1. Descriptive Statistics

Table 1 presents the descriptive statistics for the study variables. Among the academic-context indicators, the highest mean scores were observed for passion for the future profession (*M* = 8.06, *SD* = 1.81) and expected job satisfaction (*M* = 7.97, *SD* = 1.91), followed by autonomy (*M* = 7.79, *SD* = 1.88) and conditions of the educational institution (*M* = 7.04, *SD* = 2.02).

The mean SWLS total score was 25.91 (*SD* = 5.32). For the LHS dimensions, the mean scores were 45.98 (*SD* = 7.84) for Positive Sense of Life, 23.15 (*SD* = 3.91) for Life Satisfaction, 21.90 (*SD* = 3.93) for Personal Fulfilment and 16.68 (*SD* = 3.96) for joie de vivre.

Regarding burnout- and compassion-related variables, the mean scores were 23.96 (*SD* = 8.18) for ProQOL secondary traumatic stress, 31.37 (*SD* = 6.79) for ProQOL compassion satisfaction, 21.85 (*SD* = 3.60) for ProQOL burnout, 20.14 (*SD* = 9.46) for MBI emotional exhaustion, 9.61 (*SD* = 5.32) for MBI depersonalisation and 31.91 (*SD* = 7.76) for MBI personal accomplishment.

### 3.2. Correlations

Table 2 presents the Pearson correlations between indicators of life satisfaction, dimensions of happiness, burnout-related dimensions and compassion-related variables.

Overall life satisfaction (SWLS total score) showed significant associations of a small-to-moderate magnitude with the LHS dimensions, including LHS life satisfaction (*r* = 0.418, *p* < 0.001) and LHS personal fulfilment (*r* = 0.333, *p* < 0.001). ProQOL secondary traumatic stress was positively associated with ProQOL burnout (*r* = 0.328, *p* < 0.001). Higher SWLS scores were also associated with lower ProQOL burnout (r = −0.137, *p* = 0.045) and lower MBI emotional exhaustion (r = −0.178, *p* = 0.009). ProQOL compassion satisfaction was not significantly associated with ProQOL burnout (*r* = −0.107, *p* = 0.125).

MBI emotional exhaustion was strongly associated with MBI depersonalisation (r = 0.738, *p* < 0.001).

### 3.3. Differences by Gender

Independent-samples *t*-tests revealed no statistically significant gender differences in the academic-context indicators, life satisfaction, happiness, ProQOL scores or MBI dimensions examined (all *p* > 0.05).

### 3.4. Exploratory Between-Sample Observed-Score Comparisons

Welch’s independent-samples *t*-tests were conducted to compare students from Spain and Germany across the 17 indicators shown in Table 3. After applying Holm’s correction for multiple comparisons, statistically significant observed-score differences between the two samples remained for 10 indicators. German students reported higher compassion satisfaction, MBI emotional exhaustion and MBI depersonalisation. Spanish students reported higher ProQOL burnout, expected job satisfaction, passion for the future profession, MBI personal accomplishment, views on teaching, perceived conditions of the educational institution and LHS personal fulfilment. The largest standardised differences were observed for compassion satisfaction, t(479.84) = −11.79, Holm-adjusted *p* < 0.001, d = −0.96; ProQOL burnout, t(363.90) = 9.84, Holm-adjusted *p* < 0.001, d = 0.89; and expected job satisfaction, t(362.58) = 9.16, Holm-adjusted *p* < 0.001, d = 0.83. The unadjusted differences observed for secondary traumatic stress, LHS joie de vivre and specified activities did not remain statistically significant after Holm adjustment. No statistically significant observed-score differences between the two samples after Holm adjustment were found for the SWLS total score, LHS positive sense of life, LHS life satisfaction or autonomy.

## 4. Discussion

This study provides evidence on academic-context well-being indicators, global life satisfaction, multidimensional happiness and burnout-related strain among university students in Spain and Germany. The descriptive findings indicate that favourable scores on several well-being and vocational indicators coexist with emotional exhaustion and burnout-related experiences within the two university samples. This pattern is consistent with previous research showing that positive aspects of student well-being may coexist with academic stress and burnout-related strain in higher education (Lesener et al., 2020; Pascoe et al., 2020; Salmela-Aro & Read, 2017).

More specifically, favourable scores were observed on vocational indicators and perceptions of the educational environment, particularly among Spanish students, which is consistent with research showing associations between vocational and academic factors, engagement, and burnout-related experiences in university students (Vizoso Gómez & Arias Gundín, 2016; Schaufeli et al., 2002). Similarly, the emotional exhaustion and burnout-related scores observed in both samples are consistent with previous evidence documenting academic stress and burnout-related strain among university students (Lesener et al., 2020; Pascoe et al., 2020).

The correlational analyses identified associations between global life satisfaction and the LHS dimensions, as well as inverse associations with burnout-related indicators. This pattern aligns with the conceptualisation of life satisfaction as a cognitive component of subjective well-being (Diener et al., 1999). These findings are consistent with conceptualisations of subjective well-being in which global cognitive evaluations of life are related to, but distinct from, broader facets of happiness (Ryan & Deci, 2001). The low-to-moderate associations observed between the SWLS total score and the LHS dimensions are therefore consistent with partial conceptual overlap rather than equivalence between the measures.

The inverse associations observed between global life satisfaction and burnout-related indicators are consistent with findings by Gutierrez Ticona et al. (2024), who reported a negative association between academic burnout and life satisfaction in university students. Although their study was conducted in a different national context and used a different analytical approach, the convergence suggests that academic strain and global evaluations of life may be related without representing equivalent constructs. This interpretation is also compatible with the study well-being profiles identified by Vilhunen et al. (2026), whose person-oriented analysis showed different combinations of engagement, burnout and study satisfaction among university students. Although the present study examines associations between variables rather than profiles, both approaches point to heterogeneity in the configuration of positive and strain-related dimensions of student well-being.

Given the cross-sectional design, these relationships are interpreted as associations and not as evidence of protective, predictive or causal effects. Accordingly, the contribution of the present analyses lies in characterising the joint pattern of global life satisfaction, multidimensional happiness, burnout-related dimensions and compassion-related indicators, rather than in identifying independent predictors.

Taken together, these findings can also be interpreted within the Study Demands–Resources framework, which distinguishes the role of study demands and resources in relation to student burnout and engagement (Lesener et al., 2020; Salmela-Aro & Upadyaya, 2014). Because study demands and resources were not directly assessed, the framework is used here as an interpretive context rather than as a model empirically tested in the present study. Recent developments in SD-R theory further emphasise that demands and resources may have differentiated and combined relationships with student functioning (Bakker & Mostert, 2024). Accordingly, the framework helps situate the observed associations among well-being and burnout-related indicators without implying that specific academic demands or resources were tested as explanatory mechanisms in the present study. The strong positive association between MBI emotional exhaustion and depersonalisation further shows that these burnout-related dimensions co-occurred within the present sample, consistent with the multidimensional conceptualisation of burnout (Maslach & Jackson, 1981).

Furthermore, the positive association observed between secondary traumatic stress and ProQOL burnout can be interpreted in light of previous studies examining ProQOL dimensions in students undertaking helping-oriented and pre-professional training activities (Harr et al., 2014; Chachula, 2021; Hamaideh et al., 2023). Compassion satisfaction, by contrast, was not significantly associated with ProQOL burnout in the present sample. However, given the heterogeneity of the present sample, these ProQOL findings should be interpreted cautiously and should not be assumed to reflect the same experiences across all degree programmes.

Gender comparisons revealed no significant differences in any of the dimensions assessed. However, previous studies have reported gender differences in burnout among higher education students (Salmela-Aro & Read, 2017). Therefore, the absence of significant gender differences in the present study should be interpreted as a sample-specific finding rather than as evidence of equivalent well-being profiles across genders more generally.

The exploratory between-sample analyses revealed a differentiated pattern of observed-score differences across the assessed variables. These comparisons describe the statistical pattern observed in the present Spanish and German samples but should not be interpreted as evidence of invariant cross-cultural differences in the underlying constructs. The strongest difference concerned compassion satisfaction, which was higher among German students, whereas ProQOL burnout was higher among Spanish students. German students also obtained higher scores on MBI emotional exhaustion and depersonalisation, whereas Spanish students reported higher expected job satisfaction, greater passion for the future profession, higher MBI personal accomplishment, more favourable views on teaching and institutional conditions, and higher LHS personal fulfilment. By contrast, the two groups did not differ after Holm adjustment in secondary traumatic stress, LHS joie de vivre, specified activities, the SWLS total score, LHS positive sense of life, LHS life satisfaction or autonomy. These findings should be interpreted as observed-score differences between the two samples rather than as evidence of invariant cultural differences. This pattern is consistent with comparative research suggesting that student well-being may vary according to institutional, social and economic conditions across higher-education contexts (Hauschildt et al., 2021). However, these contextual factors were not directly assessed in the present study and therefore cannot be used to explain the observed between-country differences.

### Limitations and Future Research

Several limitations should be acknowledged. The use of a convenience sample and a cross-sectional design limits the generalisability of the findings and prevents causal or temporal interpretations. The sample size was not determined using an a priori power analysis, and the smaller German subgroup (*n* = 220) may have reduced sensitivity to small between-country differences. In addition, the use of self-report measures may have introduced response biases. Measurement invariance across the Spanish and German samples was not examined; therefore, the between-country comparisons reported here refer to differences in observed scores and should not be interpreted as evidence of latent or culturally invariant differences. The study also did not assess potentially relevant variables such as resilience, social support, loneliness, relationship status, financial resources, social-media use or other aspects of access and social participation. Students’ expectations, responsibilities and attitudes toward the academic environment were also not directly assessed and may be relevant when interpreting subjective perceptions of teaching and institutional conditions. In addition, the heterogeneous composition of the sample limits the extent to which ProQOL-related findings can be generalised across degree programmes, particularly because previous student applications have often focused on helping-oriented or pre-professional training contexts. In addition, the analytical scope of the study is primarily descriptive and correlational and does not estimate independent multivariable effects; consequently, the findings should not be interpreted as identifying unique predictors of student well-being or burnout-related strain.

Future studies should use more representative and multicentre samples and longitudinal designs to examine changes in student well-being and burnout-related strain over time. They should also incorporate personal and contextual resources, including social and economic conditions, and examine configural, metric and scalar invariance before drawing stronger conclusions from between-country comparisons. Further research could specifically examine ProQOL dimensions across different degree programmes and training contexts to determine whether the observed patterns vary according to the interpersonal and helping demands associated with particular fields of study.

A further avenue for research would be to examine digital-learning demands, social-media use and AI-related academic practices as contextual factors potentially relevant to student well-being and burnout (Bond et al., 2024; European Commission, 2022). These factors were not assessed in the present study and should therefore not be inferred from the observed findings.

## 5. Conclusions

This study indicates a pattern in which favourable indicators of subjective well-being coexist with burnout-related strain among university students in Spain and Germany. Global life satisfaction was positively associated with dimensions of happiness and inversely associated with burnout-related indicators, although the cross-sectional design does not permit causal interpretation. No significant gender differences were observed. After Holm adjustment for multiple comparisons, 10 of the 17 exploratory between-sample observed-score comparisons remained statistically significant. These findings refer to observed-score differences between the two samples and do not establish latent or culturally invariant differences. The pattern was domain-specific: German students reported higher compassion satisfaction and higher MBI emotional exhaustion and depersonalisation, whereas Spanish students reported higher ProQOL burnout, higher expected job satisfaction, greater passion for future professions, higher MBI personal accomplishment and more favourable scores on several academic-context indicators. These findings should be interpreted as observed-score differences between the two samples. Taken together, the findings support a multidimensional understanding of student well-being, in which global life satisfaction, academic-context evaluations and burnout-related strain represent related but distinct dimensions of students’ psychosocial experience. Future research should use representative and longitudinal designs, examine measurement invariance and incorporate additional personal and contextual factors that may contribute to student well-being.

## Figures and Tables

**Table 1:** Descriptive statistics for study variables.

Variable	*M*	*SD*
Specified activities	6.68	2.21
Autonomy	7.79	1.88
Views on teaching	6.43	2.35
Conditions of the educational institution	7.04	2.02
Passion for the future profession	8.06	1.81
Expected job satisfaction	7.97	1.91
SWLS total score	25.91	5.32
LHS—Positive Sense of Life	45.98	7.84
LHS—Life Satisfaction	23.15	3.91
LHS—Personal Fulfilment	21.90	3.93
LHS—Joie de vivre	16.68	3.96
ProQOL—Secondary traumatic stress	23.96	8.18
ProQOL—Compassion satisfaction	31.37	6.79
ProQOL—Burnout	21.85	3.60
MBI—Emotional exhaustion	20.14	9.46
MBI—Depersonalisation	9.61	5.32
MBI—Personal accomplishment	31.91	7.76

Note. *M* = mean; *SD* = standard deviation. Theoretical score ranges and scoring procedures are reported in the Instruments section.

**Table 2:** Pearson correlations among life satisfaction, happiness, burnout and compassion-related variables.

Relationship Between Variables	*r*	*p*
SWLS total score ↔ LHS—Life satisfaction	0.418	<0.001
SWLS total score ↔ LHS—Positive sense of life	0.263	<0.001
SWLS total score ↔ LHS—Personal fulfilment	0.333	<0.001
SWLS total score ↔ *LHS*—Joie de vivre	0.181	0.002
SWLS total score ↔ ProQOL—Burnout	−0.137	0.045
SWLS total score ↔ MBI—Emotional exhaustion	−0.178	0.009
ProQOL—Secondary traumatic stress ↔ ProQOL—Burnout	0.328	<0.001
ProQOL—Compassion satisfaction ↔ ProQOL—Burnout	−0.107	0.125
MBI—Emotional exhaustion ↔ MBI—Depersonalisation	0.738	<0.001

Note. Pearson correlation coefficients are reported. Only correlations relevant to the study aims are shown. SWLS = Satisfaction With Life Scale; ProQOL = Professional Quality of Life Scale; MBI = Maslach Burnout Inventory. Statistical significance was set at *p* < 0.05.

**Table 3:** Exploratory observed-score comparisons between the Spanish and German samples.

Variable	Spain *M* (*SD*)	Germany *M* (*SD*)	*t*	*df*	*p*	*p*_Holm	*d*	*g*
ProQOL—Compassion satisfaction	29.29 (6.34)	35.24 (5.86)	−11.79	479.84	<0.001	<0.001	−0.96	−0.96
ProQOL—Burnout	22.88 (2.99)	19.94 (3.85)	9.84	363.90	<0.001	<0.001	0.89	0.89
Expected job satisfaction	8.48 (1.60)	7.01 (2.07)	9.16	362.58	<0.001	<0.001	0.83	0.83
Passion for the future profession	8.44 (1.71)	7.35 (1.78)	7.42	433.44	<0.001	<0.001	0.63	0.63
MBI—Emotional exhaustion	18.17 (9.36)	23.78 (8.54)	−7.59	485.08	<0.001	<0.001	−0.62	−0.62
MBI—Depersonalisation	8.57 (5.44)	11.53 (4.50)	−7.30	524.79	<0.001	<0.001	−0.58	−0.58
MBI—Personal accomplishment	33.20 (8.08)	29.53 (6.51)	6.18	535.22	<0.001	<0.001	0.48	0.48
Views on teaching	6.76 (2.22)	5.83 (2.46)	4.67	410.93	<0.001	<0.001	0.40	0.40
Conditions of the educational institution	7.26 (2.05)	6.62 (1.90)	3.92	478.73	<0.001	<0.001	0.32	0.32
LHS—Personal fulfilment	22.31 (4.02)	21.15 (3.64)	3.67	488.17	<0.001	0.002	0.30	0.30
ProQOL—Secondary traumatic stress	24.58 (6.89)	22.81 (10.07)	2.33	332.13	0.020	0.143	0.22	0.22
Specified activities	6.55 (2.35)	6.93 (1.92)	−2.18	529.72	0.030	0.178	−0.17	−0.17
LHS—Joie de vivre	16.40 (2.95)	17.20 (5.33)	−2.06	293.02	0.040	0.201	−0.20	−0.20
SWLS total score	26.13 (5.24)	25.51 (5.46)	1.38	433.07	0.169	0.673	0.12	0.12
LHS—Positive sense of life	45.72 (8.57)	46.47 (6.26)	−1.25	571.33	0.211	0.673	−0.10	−0.10
LHS—Life satisfaction	23.10 (3.89)	23.24 (3.94)	−0.43	443.76	0.670	1.000	−0.04	−0.04
Autonomy	7.80 (1.87)	7.78 (1.91)	0.13	440.56	0.900	1.000	0.01	0.01

Note. *M* = mean; *SD* = standard deviation; *d* = Cohen’s *d*; *g* = Hedges’ *g*. Two-sided Welch’s independent-samples *t*-tests were used. *p*_Holm denotes Holm-adjusted *p* values across the 17 final comparisons. Spain n = 408; Germany n = 220. Positive *t*/*d*/*g* indicate higher scores in Spain; negative values indicate higher scores in Germany. These analyses compare observed composite scores between the two recruitment samples. Because measurement invariance was not established, they should not be interpreted as tests of latent mean differences or as evidence of invariant cross-cultural differences.

## Data Availability

The data presented in this study are available on reasonable request from the corresponding author, subject to ethical and data protection restrictions.
